# COVID-19: Dogma Over Potential for Prolonged Droplet Dispersal in Air

**DOI:** 10.3389/fpubh.2020.551836

**Published:** 2020-10-30

**Authors:** Jonathan S. West, Sarah A. M. Perryman

**Affiliations:** ^1^Biointeractions and Crop Protection, Rothamsted Research, Harpenden, United Kingdom; ^2^Computational and Analytical Sciences, Rothamsted Research, Harpenden, United Kingdom

**Keywords:** droplet dispersal, bioaerosol, aerobiology, SARS-CoV-2, COVID-19

## Introduction

A typical cough or sneeze produces thousands of different droplets, some of which are relatively large, say 0.5–3 mm in diameter and these typically travel less than a few meters but have been reported to go up to 6 m (1). Many more droplets produced are much smaller (ranging from <0.3 to 2.5 μm) (2) and as a result, just like some wind-dispersed spores and pollen, can remain suspended in air for many seconds, hours, and even days in certain conditions aided by turbulence, updrafts and thermals. Although the smallest droplets are much more numerous (97% reported to be <1 μm^3^), the volume of larger droplets exceeds that of the combined volume of smaller droplets and it is not known how many virus particles are contained in small or large droplets. There is a great deal of variation in reported droplet production between different studies [e.g., Duguid (3) reported most droplets produced from sneezes, coughs, and from talking loudly were between 25 and 75 μm, with some droplets up to 2,000 μm in diameter, while more recent studies focussing on coughing primarily report much smaller droplets to be more numerous, mostly 0.5–12 μm in diameter (4–6) and one recent study suggest that no droplets are produced over 16 μm in diameter (7)]. Some studies use the term droplet nuclei for the smallest droplets (<5 μm diameter) and “droplet” for those over 5 μm diameter (8) “that fall rapidly to the ground under gravity, and therefore are transmitted only over a limited distance (e.g., ≤1 m)” (8). We will examine this point later in this article by referring to all size categories as droplets and discussing the fall speeds of small droplets in still air and studies on plant pathogens in droplets produced from rain-splashes, which have shown dispersal of airborne droplets <1 mm in diameter exceeding several meters in moderately windy conditions (9). These intermediate-sized droplets ranging from 10 to 1,000 μm may therefore pose a risk in terms of remaining airborne for significant periods (e.g., 0.3–10 min) and could contain a relatively large source of inoculum.

In conditions of low relative humidity, evaporation of water molecules from the surface of droplets makes them ever smaller and lighter and for droplets containing virus or other constituents, such as mucus, salts and proteins, evaporation while the droplet is suspended in air, leaves the virus particles and other materials originally in the droplet, suspended in air as a smaller dry particle made up of the different constituents clumped together as one. In some cases, such desiccation and concentration of salts may denature a virus but it is plausible that some virus particles can remain active for at least short periods when dry and longer if humid conditions prevent evaporation so that the virus remains protected in a droplet. Both small and large droplets can be responsible for contaminating nearby surfaces that they fall onto, or the hands of the affected person, leading to spread of the disease by later contact with an uninfected person. This is thought to be the main route of transmission following studies on influenza (9). For this reason, advice to avoid SARS-CoV-2, the virus that causes COVID-19 disease, and more generally to avoid other infections is to wash hands frequently and avoid touching the face or close contact with another person. In addition, common advice given as a response to the COVID-19 epidemic is to keep a distance of at least 2 m from another person (USA and UK government advice). This is because coarse droplets could be projected by coughs, sneezes, and even speech to contaminate another person's face, hands, or clothes, while microscopic droplets (<10 μm in diameter) can remain in the air as an aerosol and in addition to falling onto clothes and surfaces, they could be inhaled directly by an uninfected person to infect their upper or lower respiratory tract. Clearly, the further away from a potentially infected person we stand, the more we reduce the risk of catching the infection. But how far away is a safe distance? Here, we review what factors affect the risk of infection by dispersal of droplets in air and how that can guide advice to avoid COVID-19 infection with reference to an under-reported size-range of fine spray droplets that could carry the virus.

It is often difficult to study dispersal of human pathogens in real conditions although a lot can be inferred from epidemiological studies and mathematical modeling. However, there is a wealth of literature on the dispersal of plant pathogen spores that we can use as a proxy to estimate droplet dispersal of human viruses in this case. We are not considering dispersal of dry spores or plant pollen that are adapted for long-distance dispersal, only the dispersal of water droplets that may contain fungal or oomycete spores, bacteria or virus particles. In the case of plant pathogens, these can be splashed from infected leaves, fruit or plant debris. The behavior of a water droplet once in the air is the same, whether it holds a biological particle or not and once the droplet's momentum from the initial release event has subsided and the droplet is suspended as an aerosol.

## Droplet Dispersal in Still and Moving Air

A wealth of studies has shown splash dispersal of plant pathogens in controlled conditions by dropping simulated raindrops down a shaft or tower onto an infected plant or by sampling in field conditions (10–14). Spore-containing droplets splashed from the infected plant or other media may be collected on water-sensitive paper, microscope slides or by other passive air-sampling devices arranged at different distances downwind. Just as with coughs and sneezes, different sizes of droplet are produced. They comprise relatively large droplets (1–5.5 mm diameter) that are dispersed ballistically up to a few meters [i.e., following a trajectory based on their speed of release and size, which affects their momentum; aerosolised microscopic droplets (<10 μm), which remain airborne for considerable periods (e.g., over a hour) even in still air; and intermediate-sized spray droplets (>10 μm <1 mm diameter) that form a fine spray that fall in still air but remain airborne for much longer than ballistic droplets and are able to be blown in the wind for distances up to tens of meters]. Perryman et al. (3), using a wind-tunnel in combination with a rain-tower to produce splash droplets from the surface of orange fruits infected with a fungus, found that as wind speed increased, an increasing number of fine droplets (>10 μm <1 mm diameter) were blown downwind. At the maximum wind speed investigated (7 m s^−1^), these fine droplets were detected 8 m downwind of the source and their maximum vertical position with distance downwind showed that some were still moving upwards, meaning that they were behaving as an aerosol affected by eddy diffusion (15). Evaporation of these droplets was considered to be negligible during a flight covering 8 m in <1.15 s and because the ambient conditions were relatively cool and humid (around 15°C and 70–80% relative humidity).

The fall-speed (cm per second) of a spherical object with the density of water in still air at 20°C is approximated to 0.00308 multiplied by the square of the particle diameter (in μm) (16). So, a roughly spherical droplet of 2 μm diameter would fall at 0.012 cm/s, a droplet of 20 μm diameter, would fall at around 1.2 cm/s and a particle of about 200 μm diameter (0.2 mm) would fall at 123 cm/s. In contrast to the plant pathology studies, where the kinetic force producing the splash droplets came from the falling rain drop, Lindsley et al. (2) found that most aerosol droplets produced by a person coughing were under 2.5 μm diameter, so these would fall at negligible speeds. This means that in still conditions, a fine aerosol produced from coughing could remain airborne for hours, while larger droplets (larger than the aerosol fraction) generated by a cough or sneeze from a person would fall onto nearby surfaces. Intermediate-sized droplets (>10 μm <1 mm diameter) seem to be relatively rarely produced by coughing (1, 2) but if these moved due to the turbulence caused by the force of a cough or sneeze to say 2 m height, they would then take about 30 s for a 20 μm droplet to fall back 40 cm, to where somebody 1.6 m in height might breath it in.

## Sars-CoV-2 Survival in Droplets

Factors affecting the survival and dispersal of the virus include the material the droplet is formed of (i.e., a solution of mucus, salts, and cell contents in water, and the conditions the droplet is exposed to during dispersal). The loss of water by evaporation from the surface of droplets during their flight may affect the activity (activity is used here because the virus particle itself is not a viable organism but when active can infect a living cell) or loss of activity of the infectious agent being transported because the water may partially protect the enclosed virus from desiccation and variations of temperature, and exposure to oxygen, ozone, and other chemicals. Estimates made for SARS-CoV-2 persistence of activity while suspended in air range from up to 30 min to a half-life in air of several hours (17–19). However, it is unclear whether different droplet sizes, ambient conditions (temperature and relative humidity) and droplet compositions (concentration of virus and cell contents) have been studied. A virus in a microscopic water droplet may become a free virus particle suspended in air if the water droplet around it evaporates. Such a dry and microscopic virus particle is more likely to lose activity but could remain airborne and in a dry state, may not be completely prevented from being inhaled even by a specialist facemask such as the N95 or FFP2 mask (1). The “infective dose” for SARS-CoV-2 is not known but the idea of an infective dose is really down to a combination of probabilities of infection occurring based on the percentage of virus particles that are actually active, evade immune responses and whether they end up at a potential infection site. Although the chances of infection decline as the number of active virus particles a person is exposed to reduces, in theory, just one active virus particle falling onto exactly the right receptor could still lead to infection, while a high exposure to virus, increases this risk of infection considerably.

## Discussion

Following the study of Perryman et al. (10), it is clear that effects of turbulence in moving air can cause intermediate-sized droplets (10 μm to 1 mm) to remain airborne and even disperse upwards as they travel down wind. This size-range of droplets is larger than those normally considered to form an aerosol and may have been neglected in previous medical studies but many other biological particles in this size range are known to disperse in air (e.g., uredospores of cereal rust fungi are 22 μm in aerodynamic diameter (15) and have been reported to disperse in air over continents and oceans (20), moss spores up to 40 μm diameter have been collected in the arctic having dispersed from warmer production sites (15) and grass pollen ranging from 20 to 50 μm diameter (15) is known to be primarily produced in the countryside in great amounts but is dispersed to affect people prone to hay-fever in city centers, often several Km from the nearest large sources of flowering grass). Although these and any aerosolised microscopic droplets would dilute as they disperse over distance and time, just like a plume of smoke, it is possible they could be breathed in by an unsuspecting person seconds, minutes, and even hours later. To sample very small droplets or small dry particles, it is important to use the correct air sampling devices such as wet cyclones or liquid impingers, which are designed to collect particles as small as below 1 μm (aerodynamic diameter) with good collection efficiency (21). One COVID-19 study (22) concluded that the virus wasn't airborne but had used an air sampler that directs the airflow via a perforated plate to impact onto solid culture media, and this may not collect small particles efficiently. Data for similar devices show EC_50_ (the cut-off for collection of 50% of particles) values to be over 4 μm, meaning that droplets smaller than this are increasingly less likely to be collected (21).

The persistence of activity of SARS-CoV-2 particles in airborne droplets appears to be sufficient to pose a threat. Droplets may not even require coughing because production of fine droplets containing influenza virus particles has been demonstrated simply from normal breathing (23). If subsequent dispersal of aerosolised droplets shown in plant pathogen studies pertain to aerosolised droplets containing SARS-CoV-2 virus particles, and assuming estimates of up to 1–7-h half-life (duration of activity) (18, 19) of the SARS-CoV-2 virus in typical ambient conditions are correct, logic suggests there must be potential for an aerosol-based infection route for this disease, which could occur at significant time after droplet production and also at distances downwind of an infected person in outdoor conditions. Huang (1) suggests that larger droplets may also pose a risk based on the inner surfaces of the nose acting as a potential infection site. If that is the case, the fact that many thousands of microscopic droplets are produced by a cough and with the possibility that intermediate-sized droplets can also remain in air for many seconds, traveling many meters in outdoor moving air, greater importance should be placed on using facemasks to prevent these droplets being inhaled, in addition to the current advice to wash hands regularly.

## Author Contributions

The article was written by JW and SP. All authors contributed to the article and approved the submitted version.

## Conflict of Interest

The authors declare that the research was conducted in the absence of any commercial or financial relationships that could be construed as a potential conflict of interest.
